# Report on the Disease Which Prevailed Lately in Paris under the Name of the Gripe

**Published:** 1803-05-01

**Authors:** 


**Report** *on the* **Disease** *which prevailed lately in Paris under the Name of the* **Gripe**; *communicated by our Correspondent.*

THE prefect of the department of the Seine having desired a statement to be drawn up of the symptoms and mode of treating this disease, Citizens Lapisse, Bouvier, Sedillot, and Leveille were appointed by the Society of Medicine of Paris to comply with the wishes of the Government. Of this Report the following is an Extract.

They state the disease to be a mild catarrhal fever, but liable to dangerous consequences from causes to be hereafter detailed. The exciting cause they attribute to great variations in the temperature of the air for two months previous to its appearance; to north and north easterly winds which prevailed during the nights, and to the south south-west which blew during the day; to the sudden changes of temperature, which in a few hours was observed to vary from 5 to 6 degrees above Zero to 5 or 6 below. To this they add the humidity of the autumn, which had succeeded a long-continued drought, and which they conceive rendered the animal body extremely irritable. The well known history and treatment of all the catarrhal diseases which have appeared in France since the year 1500, leave no room for practitioners to doubt as to the nature or cure of this disease. They state the general symptoms to be, uneasiness, loss of appetite for some days previous, shiverings succeeded by heat, pain in the head, particularly in the forehead above the eye-brows, sense of weight, partial sweats, white, sometimes yellowish tongue. The fever recurs at night, with remission of the symptoms towards morning; the pulse is frequent, hard, sometimes depressed, and getting stronger as the complaint advances to a crisis. The fever continues three, five, seven, twelve, fifteen days, and often longer, but its character changes. Its termination is accompanied either with changed urine or by abundant and general sweats, or by a puriform expectoration, mucous or bilious purgings, and sometimes many excretions unite to complete the crisis. The varieties in the seat of the disease they state to be, 1st. Catarrhus nasalis; in addition to the usual symptoms were observed, vertigo, acute pain in the ears, swelling of the parotid glands and of the face, sometimes painful and putting on an erysipelatous appearance. The conjunctiva was inflamed, with a flowing of tears more or less acrid, irritating tating and excoriating the cheeks, &c. Sometimes these parts became swoln, without any discharge whatever.

2d variety, Catarrhus gutturalis or quincy. To the common symptoms were superadded a slight phlogosis only of the fauces, painful sensation along the trachea, and aphonia, with a large discharge of thick mucus from the posterior fauces.

3d variety, Catarrhus bronchialis. The common symptoms of this complaint were aggravated by a dry, hard, and continued cough with constant irritation, a difficulty of respiration, stitch in the side, wandering pains similar to rheumatic, expectoration difficult, frothy, sometimes streaked with blood, hæmorrhagy from the nose, chest, and hæmorrhoids, sometimes symptomatic, sometimes critical, but in general found to relieve the pain in the head. This complaint is difficult to distinguish from peripneumony and can only be determined by the degree of violence of the symptoms. This complaint terminates by spitting or sweats; expectoration becoming more easy and the spits more thick and puriform. Sometimes this complaint is succeeded by *metastasis* or imperfect crisis, and rheumatismal affections of the extremities.

4th variety, Catarrhus suffocans. This variety was fortunately but rarely met with. Old men, or those in previous ill health, were thus attacked. It was sometimes the consequence of bad treatment, or errors committed in the regimen, and frequently carried off the patient when danger was least expected. The increase of oppression about the breast, and of the other symptoms enumerated, was suddenly followed by diminution of strength; and a sudden congestion in the lungs closed the scene. It may not be unnecessary to observe, that the varieties here marked by the reporters have seldom been found isolated, the head for the most part became first affected, and successively the throat and chest, and very frequently many of these varieties appeared at one and the same time in the same person. The last variety which they notice is an affection of the stomach and bowels, marked by a discharge of mucus, with cholic pains, and often a dysentery, which carried off the person quickly. It makes a fifth variety under the title Catarrhus intestinalis.

They remark that the complaint sometimes puts on a more inflammatory appearance, in which the usual pleuritic symptoms are more violent and better marked; this variety has been seldom met with, though they think it may become more frequent if cold and dry weather should set set in and be continued any time. The complaint has been sometimes found associated with putrid fever among the poorer classes and in hospitals. These combinations every prudent practitioner will have in view, and are sufficient to shew that every exclusive curative method is only the suggestion of ignorance or of quackery. We have more fully detailed the part of the Report which is occupied with the appearances of the disease, but do not think it equally necessary to enter into the particulars of the prognosis or treatment; the former of which has been anticipated by observing that it was of a mild nature, and only rendered dangerous by the circumstances that have been marked. The catarrh often remains after the fever has passed off; relapses are frequent, and convalescence tedious. The sediment in the urine, sweats, and expectoration, mark the crisis. The causes which tend to exasperate the complaint are, neglect in the beginning, treating it as a common catarrh by endeavouring to restore perspiration, the improper use of evacuation, blood-letting, &c. and lastly, to the variety of quack medicines with which the city is inundated. We cannot avoid making one observation, in addition to those already extracted from the Report; that is, that in every general cause of mortality, care should be taken to separate the ordinary from the unusual causes of death. The public mind is too apt to exaggerate danger, and frequently confounds what occurs every day, and unnoticed, with what is considered to be new; and in the general alarm, they forget that other complaints are no less active than ever. No person is supposed to die but of the reigning disease. And if we add to this, the number of aged and infirm who at this period of the year only wait for an *excuse* to die, or, in other words, who only want the slightest cause to carry them off, we believe that the number of people who have died of the virulence of this disease is very small indeed.

The general treatment which they point out is, to keep the patient in bed, moderately warm; to give pectoral drinks, taken warm and in small quantity; decoction of bran, light broths, inhalation of warm vapours, drinks sweetened by syrup of marsh-mallows, of diacodion if there should be a loss of rest, which is generally the case; bathing the feet, emollient clysters; at the close of the disease some restoratives aided by light tonics; the more simple the treatment the more easy and certain the convalescence. In the case of quinzy they recommend the application of leeches to the throat, emollient cataplasms to to the region of the amygdalæ; in the case of nausea or mucous congestion, 15 to 20 grains of ipecacuanha. If difficulty of breathing should come on, synapisms or blisters to the throat, employed as rubefacients, and taken off as soon as this effect is produced. In the case of peripneumony, in addition to the general treatment, they recommend leeches to the side, bladders of warm milk or water applied to the affected part, and lastly synapisms and blisters. If the tongue be foul, with bad taste, and nausea, give ipecac. as an emetic; the watery extract of opium administered at night is useful, by calming irritation, exciting perspiration, acting lightly tonic, and aiding expectoration; this should not be given at the approach of the crisis. If the spitting be suppressed, and an increase of oppression, apply blisters.

In the suffocating variety, not a moment should be lost. A derivation from the part affected must be produced by any means. Sharp drinks, the oxymel scillit. in large doses, ipecac. united with tartarized antimony, antim. wine in small spoonfuls every quarter of an hour, blisters to the legs, between the shoulders and breast. In the affection of the intestines, ipecacuanha as an emetic in the first days, emollient drinks, mucilaginous clysters, restoratives, opiates joined with ipecac. or the bark, according to circumstances. These varieties of the complaint are successfully treated by aiding the efforts of Nature.

In the Inflammatory Catarrh, it is useful to bleed during the first days, when the symptoms strongly mark its necessity. The state of the pulse before and after the evacuation should be carefully observed, and never forgetting the principle, that in all popular diseases protrastation of strength forbids its repetition. If, when this method is had recourse to, the strength becomes too much diminished, an opposite plan should be adopted. In the catarrh, complicated with putrid fever, as well as the general treatment, we should administer ipec. or ant. tart. in the first days; keep the bowels free, but so as not to lessen the powers; emollient drinks, with small portions of emetic, vermifuge medicines, exciting draughts, into which the red oxyd of sulphurated antimony enters, or oxymel scillit. camphorated juleps, synapisms, blisters, and light tonics, persevered in during convalescence.

*March* 20, 1803.

